# External validity limitations of randomized trials cited in adult critical care clinical practice guidelines: a systematic review of guidelines published from 2015 to 2020

**DOI:** 10.3389/fmed.2026.1826468

**Published:** 2026-08-26

**Authors:** Weiting Chen, Li Zhong, Cheng Zheng, Wenyun Ding, Kai Zhang, Nanxia Xuan, Lanxin Cao, Baoping Tian, Wei Cui, Pingli Wang, Zhongheng Zhang, Gensheng Zhang

**Affiliations:** 1 Department of Emergency Medicine, The First People’s Hospital of Linhai, Taizhou, Zhejiang, China; 2 Department of Surgical Intensive Care Medicine, Second Affiliated Hospital, Zhejiang University School of Medicine, Hangzhou, Zhejiang, China; 3 Department of Critical Care Medicine, First Affiliated Hospital of Huzhou Normal University, The First People’s Hospital of Huzhou, Huzhou, Zhejiang, China; 4 Department of Critical Care Medicine, Taizhou Municipal Hospital, Taizhou, Zhejiang, China; 5 Department of Respiration and Critical Care Medicine, Second Affiliated Hospital, Zhejiang University School of Medicine, Hangzhou, Zhejiang, China; 6 Department of General Medicine, Community Healthcare Center, North Bund, Shanghai, China; 7 Department of Intensive Care Medicine, Second Affiliated Hospital, Zhejiang University School of Medicine, Hangzhou, Zhejiang, China; 8 Department of Emergency Medicine and Provincial Key Laboratory of Precise Diagnosis and Treatment of Abdominal Infection, Sir Run Run Shaw Hospital, Zhejiang University School of Medicine, Hangzhou, Zhejiang, China; 9 School of Medicine, Shaoxing University, Shaoxing, Zhejiang, China; 10 Key Laboratory of Emergency and Trauma of Ministry of Education, Engineering Research Center for Hainan Biological Sample Resources of Major Diseases, The First Affiliated Hospital, Hainan Medical University, Haikou, Hainan, China; 11 Key Laboratory of Multiple Organ Failure, Ministry of Education, Zhejiang University, Hangzhou, Zhejiang, China; 12 Zhejiang Province Clinical Research Center for Emergency and Critical Care Medicine, Hangzhou, Zhejiang, China

**Keywords:** clinical practice guidelines, critical care medicine, external validity, randomized controlled trials, underrepresented populations

## Abstract

**Objective:**

Clinical practice guidelines (CPGs) support evidence-informed decision-making, but the randomized controlled trials (RCTs) cited as supporting evidence may have restricted eligibility criteria and limited external validity for clinically complex populations. This study aimed to describe exclusion criteria and underrepresented populations in RCTs cited by adult critical care CPGs published from October 2015 to October 2020.

**Design:**

Systematic review following the Preferred Reporting Items for Systematic Reviews and Meta-Analyses (PRISMA) guidelines.

**Data sources:**

PubMed and Scopus electronic databases were searched from October 9, 2015, to October 9, 2020.

**Eligibility criteria for selecting studies:**

A CPG was included if it fulfilled one of the following criteria: (1) the target population was adult critically ill patients; (2) critical care experts were involved in its development; or (3) all authors agreed that it was relevant to critical care medicine (CCM). CPGs were excluded if (1) the Grading of Recommendations Assessment, Development, and Evaluation (GRADE) framework was not used to develop the recommendations; (2) they were editorials, comments, meta-analyses, and reviews, duplicates, protocols, or irrelevant to CCM; (3) they were original studies that investigated the implementation of the CPGs; or (4) the topics specifically addressed critically ill child patients.

**Data extraction and analysis:**

Two independent reviewers extracted CPG characteristics, recommendations, RCT citations, and RCT exclusion criteria. Guideline quality was evaluated using the Appraisal of Guidelines for Research and Evaluation II (AGREE II) tool. Descriptive statistics were used for analysis.

**Results:**

Thirty-three CPGs publications were included. The overall average score for each AGREE II domain was >70%. These CPGs contained 1,134 recommendations and cited 1,231 RCT references. Only 8.7% of RCT citations had no exclusion criteria. The most frequent exclusion-related categories were age <18 years (37.9%), pregnancy (29.5%), moribund status (19.2%), surgical history (14.8%), renal impairment (14.2%), liver dysfunction (12.6%), hemodynamic instability (10.2%), traumatic brain injury (8.4%), allergy (8.2%), and coagulopathy (7.8%). Because the included guidelines primarily targeted adult patients, age <18 years should be interpreted as age-defined non-representation rather than inappropriate exclusion. After excluding this age-defined category, pregnancy, moribund status, surgical history, renal impairment, and liver dysfunction were the most frequent underrepresented categories.

**Conclusion:**

Among adult critical care CPGs published between 2015 and 2020, the RCT evidence cited in guideline recommendations frequently did not include several clinically important populations. These findings highlight the need for clinicians to consider the external validity of the underlying evidence when applying guideline recommendations to underrepresented or clinically complex patients.

## Highlights

- The review examined 33 adult critical care CPGs and 1,134 recommendations published from 2015 to 2020.
- RCTs cited by these CPGs frequently used exclusion criteria that may limit external validity.
- Pregnancy, moribund status, surgical history, renal impairment, and liver dysfunction were common underrepresented categories.
- Guideline recommendations should be applied with attention to the scope and applicability of the supporting evidence.

## 1 Introduction

Since the Evidence-Based Medicine Working Group introduced evidence-based medicine (EBM) in 1992, EBM has become a central framework for clinical research, medical education, and healthcare decision-making (1). In recent decades, medical societies worldwide have developed clinical practice guidelines (CPGs) to summarize the best available evidence and support clinical medicine in many specialties, including critical care medicine (CCM) (2–4). These guidelines aim to improve the quality of care, accelerate knowledge translation, and reduce unwarranted variations in practice (5, 6).

CPGs are important tools for evidence-informed decision-making. In CCM, they provide structured recommendations for common and life-threatening clinical problems and help clinicians integrate evidence, expertise, and patient values (7, 8). However, the applicability of guideline recommendations depends not only on the quality of the recommendations themselves, but also on the populations represented in the supporting evidence. This issue is particularly important in CCM and emergency medicine, where patients are often heterogeneous, physiologically unstable, and affected by multiple comorbidities or special clinical conditions (9, 10).

Several populations may be underrepresented in the randomized controlled trials (RCTs) that support adult critical care recommendations. During pregnancy, physiological changes affect multiple maternal organ systems and may alter cardiovascular, respiratory, renal, and pharmacological responses to critical illness and treatment (11). Children also differ substantially from adults in anatomy, physiology, disease presentation, and treatment response (12). Critically ill children may have acute disease onset, rapid progression, atypical clinical manifestations, and high mortality (13). However, because many adult CPGs are explicitly designed for adult patients, the exclusion of patients aged <18 years should be interpreted primarily as an age-defined boundary of applicability rather than as inappropriate exclusion from adult guidelines. Elderly patients represent another complex population because age-related reductions in physiological reserve, chronic disease burden, and frailty may modify both disease course and treatment effects (14). Patients with cancer are also clinically heterogeneous because of differences in tumor type, disease stage, treatment exposure, immune status, and organ dysfunction (15, 16).

The underrepresentation of these populations in RCT evidence may result from multiple factors, including trial scope, safety concerns, ethical considerations, regulatory requirements, disease heterogeneity, and attempts to reduce confounding. These factors do not necessarily indicate deficiencies in CPG development. Rather, they highlight a potential external validity gap between the populations enrolled in clinical trials and the patients encountered in real-world ICU practice. Therefore, when applying adult critical care CPG recommendations, clinicians should consider whether the supporting evidence directly represents the patient being treated or whether extrapolation is required.

Given the complexity of ICU populations and the potential mismatch between trial populations and real-world patients, it is important to identify which populations are commonly excluded or underrepresented in the RCTs cited by adult CCM CPGs. Such information may help clinicians interpret guideline recommendations more cautiously and may help researchers and guideline developers identify priorities for future studies. Therefore, this systematic review aimed to analyze the characteristics and categories of exclusion-related populations in RCTs cited by adult CCM CPGs published between 2015 and 2020, and to discuss how these external validity limitations may be considered in clinical practice and future guideline development.

## 2 Materials and methods

### 2.1 Design

We conducted this systematic review following the Preferred Reporting Items for Systematic Reviews and Meta-Analyses (PRISMA) guidelines.

### 2.2 Data sources and searches

PubMed and Scopus electronic databases were searched from October 9, 2015, to October 9, 2020. Guidelines published in English were considered eligible for inclusion. This predefined time window was selected to capture recently developed GRADE-based adult critical care CPGs at the time the review was designed. Therefore, the findings should be interpreted as reflecting CPGs published during 2015–2020 rather than the current guideline landscape. The search strategy and screening criteria were adapted from our previous work (9). The search keywords included “consensus,” “guideline,” “recommendation,” “statement,” “critical care,” “intensive care,” “ICU,” “critically ill,” “GRADE,” and “Grading of Recommendations Assessment, Development, and Evaluation.” The reference lists of the selected studies and reviews were also searched to identify additional articles (Supplementary material 1).

### 2.3 Study selection criteria

A CPG was deemed relevant to CCM if it fulfilled one of the following criteria: (1) the target population of the CPG was adult critically ill patients; (2) critical care experts were involved in the development of the CPG, or (3) all authors agreed that the CPG was relevant to CCM. CPGs were excluded if: (1) the GRADE framework was not used to develop the recommendations; (2) they were editorials, comments, meta-analyses, or reviews; (3) they were original studies that investigated the implementation of the CPGs; (4) they were protocols for the development of CPGs; (5) the topics were irrelevant to CCM; (6) the publications were duplicated; or (7) the topics specifically addressed critically ill child patients.

### 2.4 Data extraction

The full text of each included CPG was examined to identify randomized controlled trials (RCTs) cited in support of recommendations. Data were extracted for the following variables: country or region, publication year, Journal, development organization, PMID, CPG topic, number of recommendations, and number of RCT citations. Two reviewers (Zhong and Zheng) independently extracted the required data from eligible RCTs and all other data using an Excel datasheet. Any disagreement was resolved through discussion, and consensus was reached with the assistance of a third author when necessary.

Definitions of the exclusion-related categories are presented in Supplementary Table 2. Categories included age, pregnancy, hemodynamic instability, traumatic brain injury, coagulopathy, shock, elevated intracranial pressure, brain death, and other criteria directly reported in the RCTs. Because this review focused on adult CPGs, exclusion of patients aged <18 years was interpreted as age-defined non-representation rather than inappropriate exclusion of a special population. Detailed classifications are provided in the Appendix.

### 2.5 Quality assessment of the included CPGs

Guideline quality was evaluated using the Appraisal of Guidelines for Research and Evaluation II (AGREE II) tool (17). AGREE II consists of 23 items grouped into six domains and an overall assessment: scope and purpose, stakeholder involvement, rigor of development, clarity of presentation, applicability, and editorial independence. Each item is scored from 1 (strongly disagree) to 7 (strongly agree); when an AGREE II item is partially satisfied, a score of 2–6 is assigned according to the actual situation. Four evaluators—Zhong, Zheng, Chen, and Ding—were trained in advance on the AGREE II scoring criteria. The evaluators referred to the original version of the AGREE II, scored the 23 items in the guidelines, and calculated the scores for each domain. Scores for each domain and the overall evaluation were calculated as follows: four evaluators’ scores minus the minimum possible score, divided by the maximum possible score minus the minimum possible score.

### 2.6 Statistical analysis

Descriptive statistics were used to analyze the findings. Data were analyzed using IBM SPSS Statistics for Windows (version 19.0; IBM Corp., Armonk, NY, USA) to determine their frequencies (absolute and relative). Count data are expressed as rates or percentages, and measurement data are expressed as the mean ± standard deviation.

## 3 Results

### 3.1 Search results

**Figure 1:**
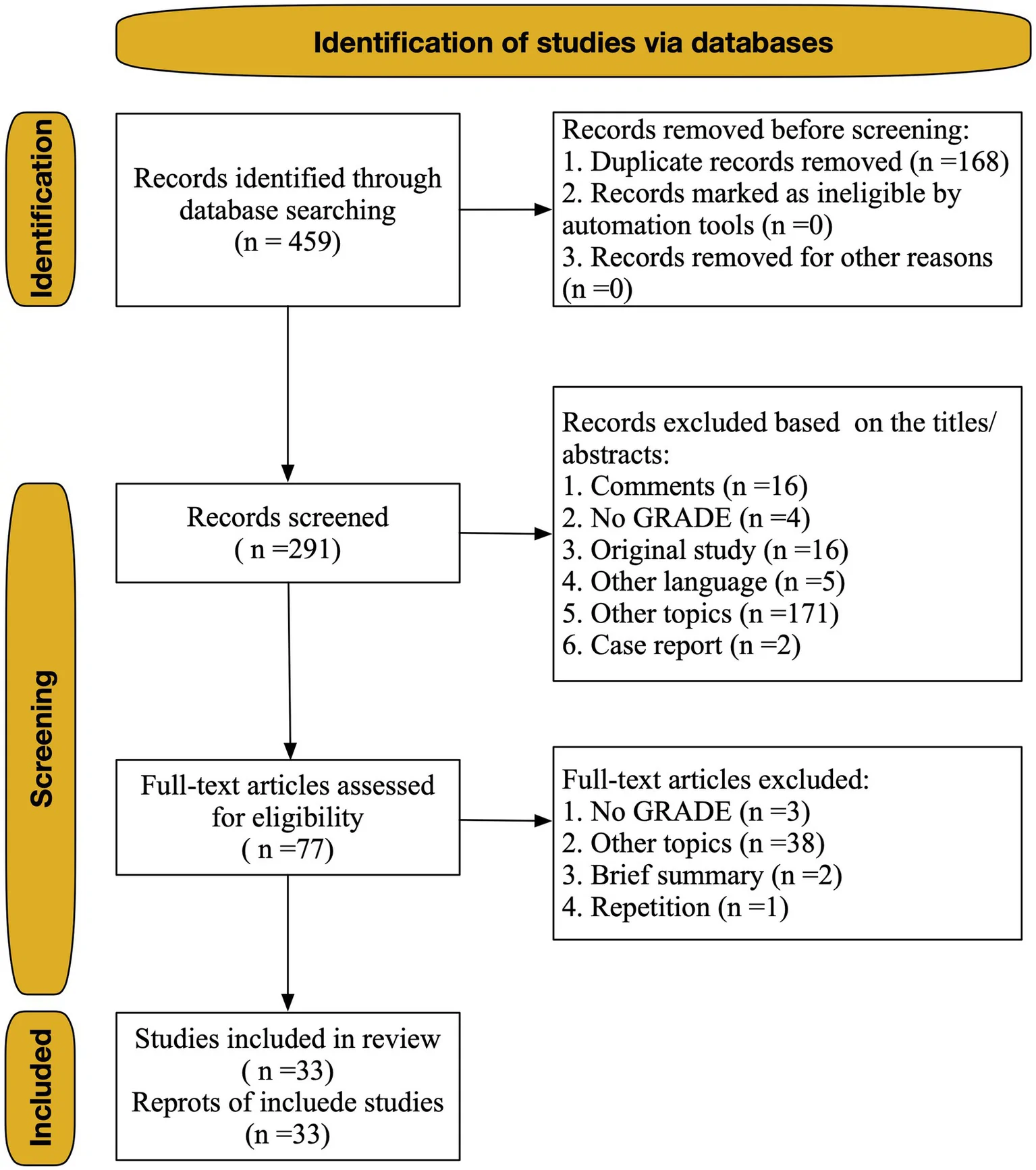
Flow diagram of study selection for adult critical care clinical practice guidelines published between 2015 and 2020. A total of 459 records were identified through database searching. After removal of 168 duplicate records, 291 records were screened by title and abstract. Seventy-seven full-text articles were assessed for eligibility, and 33 clinical practice guidelines were ultimately included in the review. Reasons for exclusion at the screening and full-text review stages are shown in the figure. CPG, clinical practice guideline; GRADE, Grading of Recommendations Assessment, Development and Evaluation.

The initial search identified 459 publications, of which 291 remained after duplicates were removed. Title and abstract screening excluded 214 records, including 16 comments or editorials, four records that did not use GRADE, 16 original studies, five records published in other languages, 171 records on irrelevant topics, and two case reports. Full-text assessment was performed for 77 publications, of which 44 were excluded (Figure 1). Consequently, 33 CPG publications were included in the current analysis, consisting of 21 categories of topics commonly associated with CCM (Supplementary Table 3) (2–4, 18–47).

### 3.2 The characteristics and AGREE II scores of the included CPGs

The included CPGs were published between 2015 and 2020. The guideline development organizations were medical societies, academic committees, or expert groups, and all used the GRADE framework. The characteristics of the included guidelines are summarized in Table 1. The included guidelines were assessed using AGREE II. The overall average score for each of the six domains was >70%, including scope and purpose, stakeholder involvement, rigor of development, clarity of presentation, applicability, and editorial independence. The specific AGREE II scores are listed in Table 2.

**Table 1:** Characteristics of the included guidelines.

References	Pubmed	Country and code	Public year	Public institution	Development organization	Reference amount
Girard et al. (37)	27762595	American	2017	American Journal of Respiratory and Critical Care Medicine	American Thoracic Society/ American College of Chest Physicians	88
Aquim et al. (18)	31967216	Brazilian	2019	Rev Bras Ter Intensiva	Associação Médica Brasileira	39
Howes et al. (19)	26417702	Canadian	2015	Resuscitation	The Canadian Association of Emergency Physicians (CAEP), the Canadian Critical Care Society (CCCS), Canadian Neurocritical Care Society (CNCCS), and the Canadian Critical Care Trials Group (CCCTG)	172
Fichtner et al. (20)	31505511	Germany	2019	Respiration	German Society of Anesthesiology and Intensive Care Medicine	63
Kane-Gill et al. (22)	28816851	American	2017	Critical Care Medicine	The American College of Critical Care Medicine	374
Murray et al. (23)	27755068	American	2016	Critical Care Medicine	The American College of Critical Care Medicine	230
Devlin et al. (24)	30113379	American, Canada, Netherlands, France, Australia,	2018	Critical Care Medicine	The Society of Critical Care Medicine	538
Chaves et al. (25)	29406956	Spanish	2020	Medicina Intensiva	Spanish Society of Infectious Diseases and Clinical Microbiology and (SEIMC) and the Spanish Society of Spanish Society of Intensive and Critical Care Medicine and Coronary Units (SEMICYUC)	289
Reintam Blaser et al. (26)	28168570	Switzerland	2017	Intensive Care Medicine	European Society of Intensive Care Medicine (ESICM)	75
Duranteau et al. (27)	29112545	France and Italy	2018	European Journal of Anaesthesiology	ESA VTE Guidelines Task Force	38
Baron et al. (28)	26609286	German	2015	Intensive Care Medicine	German Society of Anesthesiology and Intensive Care Medicine and German Interdisciplinary Association for Intensive Care and Emergency Medicine	284
Raimondi et al. (29)	28103536	American and Spain	2016	Journal of Critical Care	Federation of Societies of Critical and Intensive Therapy Medicine and Latin American Critical Care Trial Investigators Network	107
Timsit et al. (2)	32894389	French	2020	Annals of Intensive Care	French Society of Intensive Care Medicine	257
Frontera et al. (30)	26714677	American	2016	Neurocritical Care	Neurocritical Care Society and Society of Critical Care Medicine	434
Frankel et al. (41)	26468699	American, Canada, Germany, Saudi Arabia and United Kingdom	2015	Critical Care Medicine	Society of Critical Care Medicine	106
Levitov et al. (42)	27182849	American, Canada, Germany, Saudi Arabia and United Kingdom	2016	Critical Care Medicine	Critical Care Medicine	216
Pastores et al. (46)	29090327	American and France	2018	Intensive Care Medicine	Society of Critical Care Medicine (SCCM) and European Society of Intensive Care Medicine (ESICM)	15
Nanchal et al. (3)	32058387	American Canada, Saudi Arabia and France	2020	Critical Care Medicine	Society of Critical Care Medicine	190
McClave et al. (36)	26773077	American	2016	Journal of Parenteral and Enteral Nutrition	Society of Critical Care Medicine (SCCM) and American Society for Parenteral and Enteral Nutrition (A. S. P. E. N.)	480
Griffiths et al. (33)	31258917	united kingdom	2019	BMJ Open Respiratory Research	Intensive Care Society (ICS)	146
Ouellette et al. (38)	27818331	American	2017	CHEST	American College of Chest Physicians/American Thoracic Society	41
Aziz et al. (4)	32514598	United Kingdom, Saudi Arabia, Canada, American, Italy, Brazil, Belgium, France, Denmark, China	2020	Intensive Care Medicine	N/A	161
Fan et al. (34)	28459336	American	2017	American Journal of Respiratory and Critical Care Medicine	American Thoracic Society/European Society of Intensive Care Medicine/Society of Critical Care Medicine	89
Hage et al. (32)	31469325	American	2019	American Journal of Respiratory and Critical Care Medicine	American Thoracic Society	107
Guilhaumou et al. (31)	30925922	French	2019	Critical Care	French Society of Pharmacology and Therapeutics (SFPT) and the French Society of Anaesthesia and Intensive Care Medicine (SFAR)	136
Mendes et al. (47)	30843949	Portuguesa	2019	Revista Brasileira de Terapia Intensiva	Sociedade Portuguesa de Cuidados Intensivos	68
Alhazzani et al. (35)	32224769	Canada, Denmark, Saudi Arabia, American, Netherlands, China, Italy, Korea, United Kingdom, Australia	2020	Intensive Care Medicine	European Society of Intensive Care Medicine and the Society of Critical Care Medicine	222
Rhodes et al. (39)	28101605	Canada, Denmark, Saudi Arabia, American, Netherlands, China, Italy, Korea, United Kingdom, Australia, Spain, Israel, Belgium, Germany, India, Brazil, Japan,	2017	Intensive Care Medicine	Society of Critical Care Medicine (SCCM) and the European Society of Intensive Care Medicine (ESICM).	655
Cariou et al. (44)	28631089	Franch	2018	Anaesthesia Critical Care & Pain Medicine	French Intensive Care Society and French Society of Anesthesia and Intensive Care Medicine	190
Hashimoto et al. (21)	28770093	Japan	2017	Journal of Journal of Intensive Care	Japanese Society of Respiratory Care Medicine and the Japanese Society of Intensive Care Medicine	208
Madden et al. (45)	29038971	American	2017	Neurocritical Care	Neurocritical Care Society	205
Trouillet et al. (40)	29546588	French	2018	Annals of Intensive Care	French Intensive Care Society and the French Society of Anesthesia and Intensive Care Medicine	108
Vlaar et al. (43)	31912207	Canada, Denmark, Saudi Arabia, American, Netherlands, Italy, United Kingdom, France, Austria	2020	Intensive Care Medicine	European Society of Intensive Care Medicine	150

**Table 2:** AGREE II scores for included CPGs.

References	Scope and purpose (%)	Stakeholder involvement (%)	Rigor of development (%)	Clarity and presentation (%)	Applicability (%)	Editorial independence (%)	Overall assessment (%)
Girard et al. (37)	80.6	81.3	79.8	93.1	59.4	95.8	75.0
Aquim et al. (18)	76.4	37.5	48.8	75.0	14.6	37.5	50.0
Howes et al. (19)	88.9	85.4	81.0	87.5	82.3	87.5	83.3
Fichtner et al. (20)	87.5	65.6	69.0	66.7	50.0	91.7	70.8
Kane-Gill et al. (22)	84.7	69.8	80.4	79.2	56.3	85.4	79.2
Murray et al. (23)	79.2	76.0	75.6	80.6	70.8	91.7	79.2
Devlin et al. (24)	86.4	95.8	90.5	97.2	62.5	100	91.7
Chaves et al. (25)	83.3	79.2	88.1	88.9	67.7	93.8	87.5
Reintam Blaser et al. (26)	87.5	79.2	84.5	81.9	80.2	87.5	83.3
Duranteau et al. (27)	47.2	32.3	53.0	52.8	42.7	87.5	58.3
Baron et al. (28)	87.5	81.3	86.3	77.8	87.5	93.8	79.2
Raimondi et al. (29)	86.1	84.4	81.5	73.6	72.9	58.3	75.0
Timsit et al. (2)	90.3	85.4	86.9	87.5	70.8	87.5	83.3
Frontera et al. (30)	90.3	86.5	79.2	81.9	81.3	91.7	87.5
Frankel et al. (41)	93.1	87.5	87.5	83.3	79.2	77.1	79.2
Levitov et al. (42)	91.7	88.5	83.3	79.2	81.3	81.3	75.0
Pastores et al. (46)	81.9	84.4	79.8	75.0	77.1	77.1	70.8
Nanchal et al. (3)	98.6	95.8	89.3	91.7	92.7	91.7	95.8
McClave et al. (36)	80.6	89.6	85.1	77.8	81.3	87.5	58.3
Griffiths et al. (33)	70.8	67.7	59.5	75.0	64.6	60.4	79.2
Ouellette et al. (38)	95.8	91.7	84.5	90.3	89.6	95.8	87.5
Aziz et al. (4)	90.3	81.3	85.7	86.1	84.4	95.8	83.3
Fan et al. (34)	91.7	85.4	83.3	80.6	80.2	91.7	79.2
Hage et al. (32)	91.7	85.4	79.8	80.6	83.3	93.8	75.0
Guilhaumou et al. (31)	84.7	80.2	83.9	83.3	75.0	91.7	70.8
Mendes et al. (47)	63.9	22.9	46.4	77.8	29.2	20.8	37.5
Alhazzani et al. (35)	93.1	79.2	81.5	84.7	81.3	87.5	75.0
Rhodes et al. (39)	91.7	85.4	86.9	88.9	89.6	91.7	83.3
Cariou et al. (44)	83.3	65.6	69.0	81.9	66.7	87.5	70.8
Hashimoto et al. (21)	87.5	83.3	77.4	77.8	74.0	83.3	66.7
Madden et al. (45)	75.0	67.7	59.5	70.8	58.3	75.0	58.3
Trouillet et al. (40)	87.5	71.9	57.7	77.8	68.8	79.2	66.7
Vlaar et al. (43)	83.3	84.4	85.1	77.8	84.4	89.6	70.8
	84.6 (9.8)	76.9 (16.8)	77.3 (12.3)	80.7 (8.3)	70.9 (17.3)	83.3 (16.8)	74.7 (12.1)

### 3.3 Exclusion-related categories in RCTs cited by CPGs

**Figure 2:**
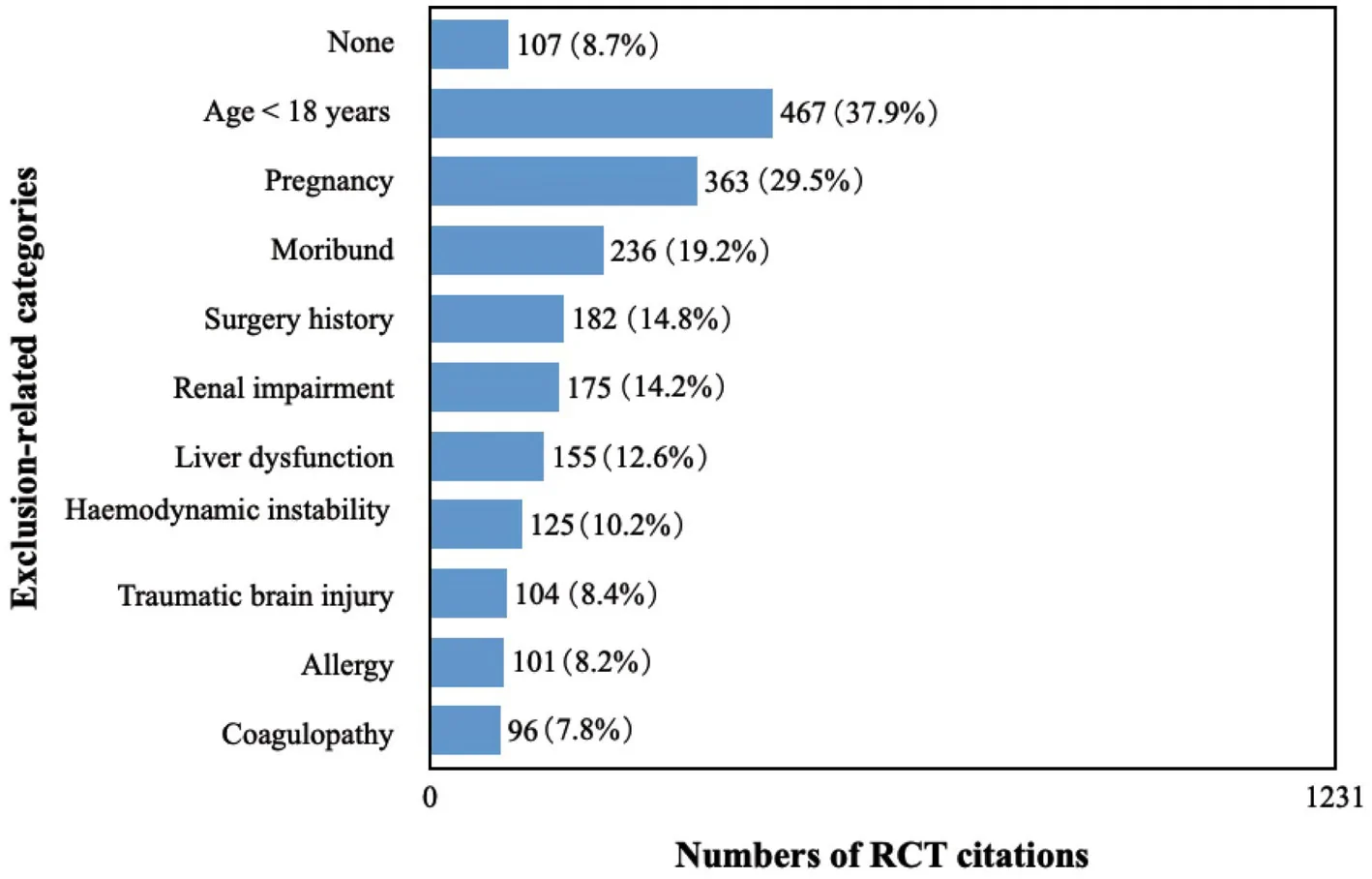
Top exclusion-related categories in RCTs cited by adult critical care clinical practice guidelines. The figure shows the most frequent exclusion-related categories identified in 1,231 randomized controlled trial citations supporting the included adult critical care guideline recommendations. Values are presented as the number and percentage of RCT citations in which each exclusion-related category was reported. Categories were not mutually exclusive, because a single RCT could report more than one exclusion criterion. RCT, randomized controlled trial; CPG, clinical practice guideline.

There were 6,481 references in the 33 CPG publications. After excluding 5,233 non-randomized records, 1,248 records remained. After duplicate removal, 1,031 full-text articles were assessed for eligibility. Further full-text screening excluded 26 articles because 12 were not RCTs and the full text of 14 articles could not be obtained. A total of 1,005 unique RCTs were included in the final analysis (Supplementary Figure 4). Among them, 151 (15.0%) RCTs were cited more than once. The total number of RCT citations was 1,231. Only 107 of 1,231 RCT citations (8.7%) had no exclusion criteria. The top ten exclusion-related categories among the 1,231 RCT citations were age <18 years (37.9%; *n* = 467), pregnancy (29.5%; *n* = 363), moribund (19.2%; *n* = 236), surgical history (14.8%; *n* = 182), renal impairment (14.2%; *n* = 175), liver dysfunction (12.6%; *n* = 155), hemodynamic instability (10.2%; *n* = 125), traumatic brain injury (8.4%; *n* = 104), allergy (8.2%, *n* = 101), and coagulopathy (7.8%, *n* = 96) (Figure 2).

**Figure 3:**
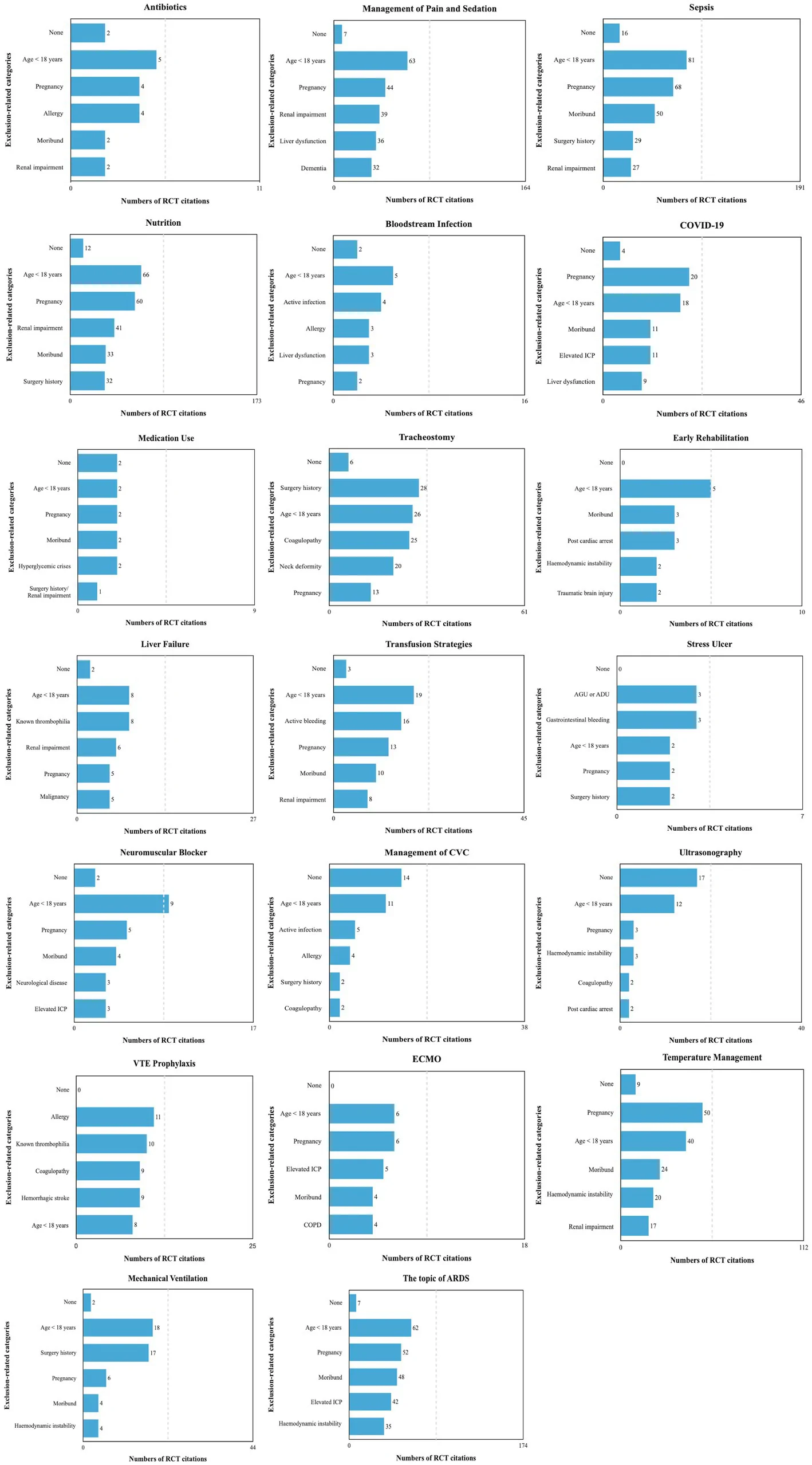
Distribution of top exclusion-related categories across adult critical care guideline topics. The figure presents the most common exclusion-related categories reported in RCT citations for each included adult critical care clinical practice guideline topic published between 2015 and 2020. The x-axis indicates the number of RCT citations, and the y-axis indicates exclusion-related categories. Categories were not mutually exclusive, because a single RCT could contribute more than one exclusion criterion. RCT, randomized controlled trial; ARDS, acute respiratory distress syndrome; ECMO, extracorporeal membrane oxygenation; CVC, central venous catheter; VTE, venous thromboembolism; ICP, intracranial pressure; HI, hemodynamic instability; CA, cardiac arrest; TBI, traumatic brain injury; COPD, chronic obstructive pulmonary disease; GIB, gastrointestinal bleeding; ASU/ADU, anti-stress ulcer or acid-dependent ulcer; AMI, acute myocardial infarction.

**Figure 4:**
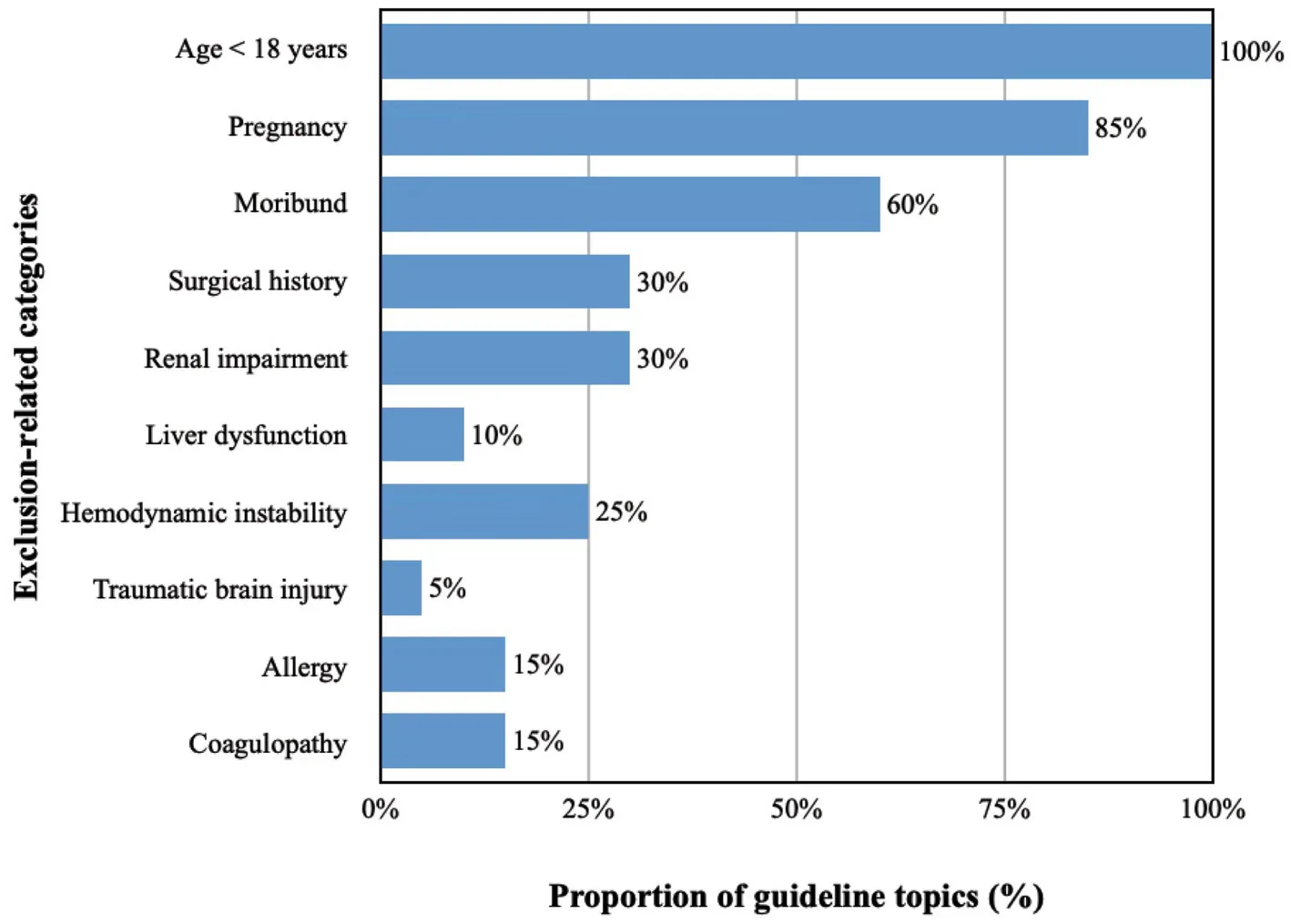
Frequency of top exclusion-related categories across adult critical care guideline topics. The figure shows the proportion of adult critical care guideline topics in which each exclusion-related category appeared among the top exclusion-related categories of RCT citations. Because the included clinical practice guidelines primarily targeted adult critically ill patients, the category “age <18 years” should be interpreted as an age-defined applicability boundary rather than inappropriate exclusion of pediatric patients. CPG, clinical practice guideline; RCT, randomized controlled trial.

The 33 CPG publications were categorized into 21 topics, including antibiotics (11 RCT citations from two CPGs) (31, 32), acute respiratory distress syndrome (ARDS; 174 RCT citations from three CPGs) (21, 33, 34), bloodstream infection (16 RCT citations from one CPG) (25), coronavirus disease 2019 (46 RCT citations from two CPGs) (4, 35), liver failure (27 RCT citations from one CPG) (3), extracorporeal membrane oxygenation (18 RCT citations from one CPG) (20), early rehabilitation (10 RCT citations from one CPG) (18), neuromuscular blocker use (17 RCT citations from one CPG) (23), management of central venous catheters (38 RCT citations from one CPG) (2), venous thromboembolism prophylaxis (25 RCT citations from two CPGs) (27, 30), nutrition (173 RCT citations from two CPGs) (26, 36), mechanical ventilation (44 RCT citations from two CPGs) (37, 38), sepsis (191 RCT citations from one CPG) (39), tracheostomy (61 RCT citations from two CPGs) (29, 40), ultrasonography (40 RCT citations from two CPGs) (41, 42), transfusion strategies (45 RCT citations from one CPG) (43), temperature management (112 RCT citations from three CPGs) (19, 44, 45), management of pain and sedation (164 RCT citations from two CPGs) (24, 28), medication use (nine RCT citations from one CPG) (22), critical illness-related corticosteroid insufficiency (three RCT citations from one CPG) (46), and stress ulcers prophylaxis (seven RCT citations from one CPG) (47) (Supplementary Table 3). Critical illness-related corticosteroid insufficiency was not analyzed because only three RCT citations were available for this topic. Twenty topics were therefore included in the final topic-level analysis. The top five exclusion-related categories for each topic are shown in Figure 3. Among these 20 topics, age <18 years appeared among the top five categories in 100% (20/20) of topics, pregnancy in 85% (17/20), moribund status in 60% (12/20), surgical history in 30% (6/20), renal impairment in 30% (6/20), and hemodynamic instability in 25% (5/20). Because the included CPGs primarily targeted adult patients, the age <18 years category should be interpreted as a boundary of applicability rather than direct evidence of inappropriate exclusion. Other exclusion-related categories across the 20 topics are shown in detail in Figure 4.

## 4 Discussion

This systematic review of adult CCM CPGs published from 2015 to 2020 found that RCTs cited in guideline recommendations commonly used eligibility criteria that may restricted the applicability of their findings to broader ICU populations. Only 8.7% of the RCT citations had no exclusion criteria. The most frequent exclusion-related categories were age <18 years, pregnancy, moribund status, surgical history, renal impairment, liver dysfunction, hemodynamic instability, traumatic brain injury, allergy, and coagulopathy. Importantly, these findings should not be interpreted as evidence that CPGs themselves inappropriately exclude these populations. Rather, they identify external validity boundaries in the RCT evidence base that supports adult critical care recommendations.

RCTs remain a major source of evidence for evaluating the efficacy and safety of interventions. Randomization reduces selection bias and confounding, thereby strengthening internal validity. However, strict eligibility criteria can also reduce external validity, particularly in CCM, where real-world patients often have multiple comorbidities, organ dysfunction, frailty, pregnancy, postoperative status, or unstable physiology. Guideline panels frequently need to translate trial evidence to patients who differ from those enrolled in RCTs. Therefore, clinicians should consider not only the strength and direction of a recommendation, but also whether the patients enrolled in the supporting trials resemble the patient being treated (48). This distinction is important because CPGs are intended to support evidence-informed decision-making rather than dictate management for every individual patient.

The finding that age <18 years appeared across all topics requires cautious interpretation. Because this review specifically included adult CPGs and excluded pediatric critical care guidelines by design, age-based exclusion primarily reflects the intended scope of adult guidelines rather than inappropriate exclusion of children from adult CPGs. Nevertheless, age-defined non-representation remains clinically relevant. Globally, there are an estimated 1.2 million cases of childhood sepsis per year (49). More than 4% of all inpatients aged <18 years and approximately 8% of pediatric ICU admissions in high-income countries have sepsis (50–52). Mortality rates in children with sepsis vary from 4 to 50% (50, 51), and many deaths occur within the first 48–72 h of refractory shock and multiple organ dysfunction syndrome (53–56). Nutritional disorders are also common among critically ill hospitalized children, with reported malnutrition rates range from 10 to 24% and associated with morbidity, mortality, and poor prognosis (57, 58). In our analysis, age <18 years appeared as an exclusion-related category in several high-frequency topics, including sepsis and nutrition. These findings should therefore be interpreted as evidence of an age-defined boundary of applicability, rather than as a criticism of adult guideline scope. This boundary should be recognized when clinicians consider guideline applicability in adolescents, transition-age patients, or clinical scenarios in which pediatric and adult critical care evidence may not align.

Pregnancy was the most frequent exclusion-related category after age-defined non-representation was set aside. The main causes of ICU admissions during pregnancy include hypertensive disorders, obstetric hemorrhage, sepsis, and infections (59). Other reasons for ICU admission included trauma, respiratory disease, cardiovascular disease, diabetic ketoacidosis, gastrointestinal, overdose or poisoning, and neurological diseases. Obstetric patients account for a small proportion of overall ICU admissions; for example, the proportion of obstetric patients among all ICU admissions in Japan has been reported to be 0.41% (60). However, pregnancy-related ICU admissions often involve severe illness and may have catastrophic consequences for maternal outcomes (61). Sepsis and other infections are important causes of maternal critical illness (59), and obstetric sepsis remains a major cause of maternal morbidity and mortality in developed countries (62–64). Similarly, ARDS is a clinically important condition during pregnancy. A previous study in the United States found that 33% of maternal deaths between 1998 and 2009 were associated with ARDS (65), and pregnant women may be at increased risk of ARDS and mechanical ventilation compared with non-pregnant women (66). In our analysis, pregnancy frequently appeared among exclusion-related categories in topics such as sepsis and ARDS. This does not necessarily mean that guideline developers ignored pregnant patients, but it indicates that the RCT evidence underlying adult CCM recommendations may provide limited direct support for applying those recommendations to pregnancy-related critical illness.

Renal impairment was also a frequent exclusion-related category. Because many RCTs did not clearly distinguish acute kidney injury (AKI) from chronic kidney disease in their exclusion criteria, the present review categorized both under renal impairment. AKI is estimated to occur in approximately 7–18% of hospitalized patients, and approximately 50% of these patients are admitted to the ICU. AKI is strongly associated with morbidity and mortality, and it has been estimated that approximately two million people die from AKI worldwide each year (67, 68). Approximately 5–10% of patients with AKI require renal replacement therapy during ICU admission (69). Survivors of AKI are also at increased risk of chronic kidney disease and end-stage renal disease, which carry high personal, social, and economic burdens (70). Therefore, when RCTs exclude patients with renal dysfunction, the applicability of guideline recommendations to a substantial proportion of ICU patients may become uncertain, especially for interventions involving drug dosing, fluid therapy, hemodynamics, nutrition, or anticoagulation.

The underrepresentation identified in this review is not caused by guidelines alone. It often reflects upstream trial design choices, safety concerns, ethical requirements, disease scope, regulatory considerations, and attempts to reduce clinical heterogeneity. For some conditions, trials involving pregnant patients, children, moribund patients, or patients with severe organ dysfunction may be difficult to design and implement. Historically, ethical concerns have also limited enrollment of vulnerable populations in interventional research. However, excessive protection may unintentionally reduce the direct evidence available to guide care for these groups (71, 72). Researchers, ethics committees, guideline developers, and clinicians should therefore continue to seek a balance between participant protection and fair evidence generation. Persistent underrepresentation creates uncertainty when guideline recommendations are applied to real ICU patients, and this uncertainty should be explicitly acknowledged in both guideline development and bedside decision-making.

Several approaches may help bridge the gap between guideline evidence and complex clinical practice. First, guideline panels could describe more explicitly the populations represented in the supporting trials and the populations for whom extrapolation is uncertain. This would help clinicians understand whether a recommendation is directly supported by trial evidence or relies on indirect evidence and clinical judgment. Second, multidisciplinary team-based decision-making remains important when guideline applicability is uncertain. The advantages of MDT consultation include rapid comprehensive assessment and development of individualized therapeutic strategies (73). For example, when obstetric patients require ICU admission, decisions should be made collaboratively among critical care specialists, obstetricians, anesthesiologists, neonatologists, and other relevant specialists (11, 74). Third, observational studies and real-world evidence may provide useful complementary information, especially when RCT evidence is limited or ethically difficult to obtain. For example, in pregnant patients with COVID-19 pneumonia, lung ultrasound and chest CT have shown comparable accuracy for assessing pulmonary involvement and predicting oxygen demand (75). Hosten et al. (76) also reported that methylprednisolone therapy was associated with pregnancy prolongation and improved maternal and neonatal outcomes in patients with HELLP syndrome.

Although such observational studies do not provide the same level of causal inference as RCTs, they can offer clinically meaningful information for populations underrepresented in trials.

Real-world studies may also help address the external validity limitations of RCT-based recommendations. In clinical practice, patients are heterogeneous in disease severity, comorbidity burden, physiological reserve, and treatment response. Therefore, CPGs based primarily on RCT evidence may not be directly applicable to every patient encountered in the ICU (77). Compared with RCTs, RWS differ in study settings, populations, methods, and intervention requirements. They are conducted in real clinical practice settings and usually impose fewer restrictions on subject selection. As a result, RWS may include patients who are commonly excluded from RCTs and may generate evidence that is more representative of routine ICU practice (78, 79). Real-world data can help clarify the gap between guideline recommendations and bedside practice, support future guideline updates, and identify patient subgroups requiring dedicated prospective studies.

Although the primary analysis of the present review was restricted to adult CCM CPGs published between 2015 and 2020, recent adult critical care guideline updates further support the continuing relevance of external validity and individualized application of recommendations (80–84). The 2026 Surviving Sepsis Campaign adult guidelines focus on adult patients with sepsis or septic shock and include 129 statements, including 46 new statements, while emphasizing that recommendations should support clinicians within specific clinical contexts (80). Similarly, the 2023 ESICM ARDS guidelines are explicitly limited to adult patients and non-pharmacological respiratory support strategies, use PRISMA and GRADE methods, and formulate 21 recommendations (81). The 2023 ESPEN ICU nutrition guideline also notes that increasing comorbidity burden and heterogeneity among adult ICU patients may reduce the external validity of recommendations, which should be used to support individualized decision-making (82). More recent examples further illustrate the same issue. The 2024 ESICM guideline on fluid therapy in adult critically ill patients provides evidence-based recommendations on the choice of resuscitation fluids, but its adult ICU scope and the need to tailor fluid decisions to clinical context again underscore the importance of cautious extrapolation to patients who differ from the populations represented in the supporting trials (83). The 2025 SCCM focused update of the PADIS guideline for adult ICU patients also expands the 2018 PADIS guideline while issuing several conditional recommendations and acknowledging areas where recommendations could not be made because of insufficient or low-certainty evidence (84). These recent guidelines were selected as illustrative examples because they correspond to high-frequency or clinically central topics in adult critical care, rather than as part of a comprehensive post-2020 guideline update. They were not included in our formal quantitative analysis and should not be interpreted as a systematic update. Nevertheless, they support the continuing importance of evaluating whether guideline recommendations can be safely and appropriately extrapolated to populations underrepresented in the underlying evidence base.

Overall, the present study highlights an important but often underrecognized issue in evidence-based critical care. Adult CCM CPGs summarize the best available evidence and remain essential tools for clinical decision-making. However, the RCTs supporting their recommendations often have eligibility criteria that limit direct applicability to some complex ICU populations. Recognizing these external validity boundaries does not weaken the value of guidelines. Instead, it promotes more careful, transparent, and individualized use of guideline recommendations in patients who differ from those enrolled in the supporting trials.

## 5 Strengths and limitations

The present review has several strengths. It used a systematic search strategy, dual-reviewer extraction, and AGREE II assessment to evaluate the quality of included CPGs. It also examined a large evidence base, including 33 adult CCM CPG publications, 1,134 recommendations, and 1,231 RCT citations. Rather than assessing only the guideline recommendations themselves, this review focused on the RCT evidence cited by these guidelines and quantified the exclusion-related criteria that may affect external validity. This approach provides a practical framework for clinicians, researchers, and guideline developers to consider the applicability of trial evidence to underrepresented or clinically complex ICU populations.

This study also has several limitations. First, only CPGs developed using the GRADE framework were included. Although GRADE is widely used and transparent, excluding non-GRADE guidelines may have introduced selection bias and limited the scope of the review (85, 86). Second, the search was limited to PubMed and Scopus. Some CPGs may be published only on professional society websites, guideline repositories, or governmental platforms and may therefore have been missed. Third, the search ended in October 2020. Thus, the findings should be interpreted as a systematic review of adult CCM CPGs published during 2015–2020 and should not be presented as a full description of the current 2026 guideline landscape. Although several recent guideline updates from 2023 to 2026 were discussed narratively to provide contemporary context, they were selected as illustrative examples because they corresponded to high-frequency or clinically central adult critical care topics, rather than through a comprehensive post-2020 search. These recent examples were not included in the formal quantitative analysis. A systematic update of post-2020 guidelines would require new screening, AGREE II assessment, extraction of cited RCTs, and reclassification of exclusion criteria. Fourth, the exclusion criteria reported in some CPGs were not sufficiently detailed, which may have led to misclassification or incomplete categorization of certain exclusion-related criteria. Fifth, meta-analyses and systematic reviews were not included as source documents for RCT identification. Therefore, some relevant RCTs that were only summarized in evidence syntheses but not directly cited by the included CPGs may have been missed.

## 6 Conclusion

Among adult CCM CPGs published from 2015 to 2020, the RCTs cited in guideline recommendations frequently used exclusion criteria that limited direct applicability to several clinically important populations. Age <18 years mainly reflected the adult scope of the included guidelines, whereas pregnancy, moribund status, surgical history, renal impairment, liver dysfunction, hemodynamic instability, traumatic brain injury, allergy, and coagulopathy represented common underrepresented or clinically complex categories in the supporting trial evidence. Clinicians should apply CPG recommendations with attention to the characteristics of the supporting trial populations and should integrate indirect evidence, observational data, multidisciplinary judgment, and patient-specific risk–benefit assessment when treating patients who were underrepresented in the evidence base.

## Data Availability

The original contributions presented in the study are included in the article/Supplementary material, further inquiries can be directed to the corresponding authors.

## Supplementary material

Supplementary Material 1 Search strategies used in PubMed and Scopus.

Supplementary Table 2 Classification definitions for the exclusion criteria.

Supplementary Table 3 Categories of topics associated with critical care medicine (CCM).

SUPPLEMENTARY FIGURE 4 Flow diagram of RCT citation screening and selection from the 33 included clinical practice guidelines. A total of 6,481 references were identified from the 33 CPGs. After exclusion of 5,233 non-randomized records, 1,248 records remained. After duplicate removal, 1,031 full-text articles were assessed for eligibility. Twenty-six articles were excluded, including 12 non-randomized studies and 14 articles for which the full text could not be obtained, leaving 1,005 unique RCTs for the final analysis. CPG, clinical practice guideline; RCT, randomized controlled trial.
