# Supplementary material for: External validity limitations of randomized trials cited in adult critical care clinical practice guidelines: a systematic review of guidelines published from 2015 to 2020
Source: Front Med (Lausanne). 2026 Aug 26;13:1826468. doi: 10.3389/fmed.2026.1826468 (PMC13557104; doi:10.3389/fmed.2026.1826468)
Supplement: Supplementary file 1 [file Table_1.docx]

**Searching strategy in databases:**

**PubMed**

**(consensus[Title] OR guideline[Title] OR guidelines[Title] OR recommendation[Title]**

**OR recommendations[Title] OR statement[Title]) AND (critical care[Title/Abstract]**

**OR intensive care[Title/Abstract] OR ICU[Title/Abstract] OR critically**

**ill[Title/Abstract]) AND (GRADE OR Grading of Recommendations Assessment,**

**Development, and Evaluation)**

**scopus**

**( ALL ( " GRADE" ) OR ALL ( " Grading of Recommendations Assessment,**

**Development, and Evaluation" ) ) AND ( TITLE ( "guideline" ) OR**

**TITLE ( "guidelines" ) OR TITLE ( "recommendation" ) OR**

**TITLE ( "recommendations" ) OR TITLE ( "consensus" ) OR TITLE ( "statement" ) )**

**AND ( TITLE-ABS-KEY ( "intensive care" ) OR TITLE-ABS-KEY ( "critical care" )**

**OR TITLE-ABS-KEY ( "ICU" ) OR TITLE-ABS-KEY ( "critically ill" ) )**
