# Supplementary material for: External validity limitations of randomized trials cited in adult critical care clinical practice guidelines: a systematic review of guidelines published from 2015 to 2020
Source: Front Med (Lausanne). 2026 Aug 26;13:1826468. doi: 10.3389/fmed.2026.1826468 (PMC13557104; doi:10.3389/fmed.2026.1826468)
Supplement: Supplementary file 2 [file Table_2.docx]

**Table .** The classification definitions for the exclusion criteria

| Excluded objects | Definition |
| --- | --- |
| Active infection | Active infection is defined as an infection of any system, or the article gives the term directly. |
| Surgery | Surgery is defined as a known history of surgery. |
| Allergy | Allergy is defined as a known history of allergy to anything, including but not limited to heparin, midazolam, propofol, eggs, egg products, plasma, vitamin K, morphine, paracetamol, enteral formula. |
| Renal impairment | Renal impairment is defined as acute kidney injury or Chronic renal failure. |
| Others | Other classifications are available directly from the article |
