# Supplementary material for: External validity limitations of randomized trials cited in adult critical care clinical practice guidelines: a systematic review of guidelines published from 2015 to 2020
Source: Front Med (Lausanne). 2026 Aug 26;13:1826468. doi: 10.3389/fmed.2026.1826468 (PMC13557104; doi:10.3389/fmed.2026.1826468)
Supplement: Supplementary file 3 [file Table_3.docx]

Table. Categories of topics associated with CCM

| **Journal** | **Journal topic** | **PMID** | **Year** | **The numbers of recommend** | **Included RCTs** | **Total number** |
| --- | --- | --- | --- | --- | --- | --- |
| Crit Care | Antibiotics | 30925922 | 2019 | 21 | 10 | 136 |
| Am J Respir Crit Care Med | Antibiotics | 31469325 | 2019 | 14 | 2 | 107 |
| J Intensive Care | Acute Respiratory Distress Syndrome | 28770093 | 2017 | 13 | 115 | 208 |
| BMJ Open Respir Res | Acute Respiratory Distress Syndrome | 31258917 | 2019 | 10 | 35 | 146 |
| Am J Respir Crit Care Med | Acute Respiratory Distress Syndrome | 28459336 | 2017 | 6 | 24 | 89 |
| Med Intensiva | Bloodstream infection | 29406956 | 2018 | 104 | 16 | 289 |
| Intensive Care Med | Critical Illness-related Corticosteroid Insufficiency | 29090327 | 2018 | 5 | 3 | 15 |
| Crit Care Med | COVID-19 | 32224769 | 2020 | 54 | 44 | 222 |
| Intensive Care Med | COVID-19 | 32514598 | 2020 | 45 | 2 | 161 |
| Rev Bras Ter Intensiva | Early rehabilitation | 31967216 | 2019 | 6 | 10 | 39 |
| Respiration | Extracorporeal Membrane Oxygenation | 31505511 | 2019 | 22 | 18 | 63 |
| Crit Care med | Liver Failure | 32058387 | 2020 | 30 | 27 | 190 |
| Ann Intensive Care | Management of central venous catheters | 32894389 | 2020 | 33 | 38 | 257 |
| Ger Med Sci | Management of Pain and Sedation | 26609286 | 2015 | 144 | 53 | 284 |
| Crit Care Med | Management of Pain and Sedation | 30113379 | 2018 | 71 | 113 | 538 |
| Crit Care Med | Medication Use | 28816851 | 2017 | 38 | 9 | 374 |
| Am J Respir Crit Care Med | Mechanical Ventilation | 27762595 | 2017 | 4 | 23 | 88 |
| Chest | Mechanical Ventilation | 27818331 | 2017 | 3 | 23 | 41 |
| Crit Care med | Neuromuscular blocker | 27755068 | 2016 | 10 | 17 | 230 |
| JPEN | Nutrition | 26773077 | 2016 | 94 | 162 | 480 |
| Intensive Care Med | Nutrition | 28168570 | 2017 | 23 | 16 | 75 |
| Intensive Care Med | Sepsis | 28101605 | 2017 | 93 | 196 | 655 |
| Rev Bras Ter Intensiva | Stress ulcer | 30843949 | 2019 | 6 | 7 | 68 |
| Resuscitation | Temperature management | 26417702 | 2016 | 48 | 10 | 172 |
| Anaesth Crit Care Pain Med | Temperature management | 28688998 | 2018 | 30 | 47 | 190 |
| Neurocrit Care | Temperature management | 29038971 | 2017 | 30 | 55 | 205 |
| Journal of Critical Care | Tracheostomy | 28103536 | 2016 | 20 | 33 | 107 |
| Anaesth Crit Care Pain Med | Tracheostomy | 29559211 | 2018 | 18 | 28 | 108 |
| Intensive Care Med | Transfusion strategies | 31912207 | 2020 | 16 | 46 | 150 |
| Crit Care Med | Ultrasonography | 26468699 | 2015 | 23 | 39 | 106 |
| Crit Care Med | Ultrasonography | 27182849 | 2016 | 43 | 2 | 216 |
| Neurocrit Care | Venous thromboembolism prophylaxis | 26714677 | 2016 | 49 | 22 | 434 |
| Eur J Anaesthesiol | Venous thromboembolism prophylaxis | 29112545 | 2018 | 8 | 3 | 38 |
